# Integrating portable qPCR and image recognition to combat illegal trade in sharks and rays

**DOI:** 10.1038/s41598-025-22370-y

**Published:** 2025-11-04

**Authors:** Diego Cardeñosa, Zhuang Luo, Kyumin Lee, Emma Aitken, Maria A. Herrera, DeEtta Mills, John Carlson, Gavin Naylor

**Affiliations:** 1 Department of Biological Sciences, Florida International University, North Miami, Florida USA; 2 Worcester Polytechnic Institute, Worcester, MA 01609 USA; 3 National Marine Fisheries Service, Panama City Beach, FL 32408 USA; 4 International Shark Attack File (ISAF), Florida Museum of Natural History, University of Florida, Gainesville, FL USA

**Keywords:** HRM assay, CITES, Enforcement, Shark conservation, International trade, Computational biology and bioinformatics, Genetics

## Abstract

Illegal trade in sharks and rays continues to undermine global conservation efforts, with enforcement often hampered by the inability to identify products to the species level. Here, we present a portable, cost-effective High-Resolution melt (HRM) assay for rapid DNA-based identification of elasmobranch species in trade. Using a reference library of 669 vouchered tissue samples collected from field operations and international market surveys, we validated the assay’s capacity to accurately differentiate at least 55 shark and ray species based on melt curve profiles, including 38 species listed under the Convention on International Trade in Endangered Species of Wild Fauna and Flora. Automated image classification enabled high-throughput identification with 99.2% accuracy. The assay yields results within two hours at a per-sample cost of $1.50, and is compatible with portable qPCR platforms, making it suitable for on-site applications. This approach represents a scalable molecular enforcement tool that can empower local authorities to monitor trade more effectively, support compliance with international regulations, and enhance global efforts to combat wildlife trafficking.

**Supplementary Information:**

The online version contains supplementary material available at 10.1038/s41598-025-22370-y.

## Introduction

Crimes against wildlife are now considered the fourth most lucrative form of illegal activity, with an estimated annual value of $20 billion year^1^. To combat these crimes, law enforcement faces many challenges, from disrupting activities in the field to securing effective prosecutions. A critical barrier occurs early in the interdiction process: the timely identification of prohibited or regulated species. Failing to quickly distinguish traded wildlife products at the species level hampers subsequent investigative steps, reduces enforcement capacity, undermines the effectiveness of Multilateral Environmental Agreements, and ultimately allows wildlife crimes to go unpunished.

The Convention on International Trade in Endangered Species of Wild Fauna and Flora (CITES) serves as a key instrument to regulate trade in threatened wildlife, including sharks and rays, which face severe conservation pressures due to overfishing and international trade in their fins and other body parts^2,3^. Large-scale surveys of shark fin retail markets in Hong Kong, the world’s largest hub, have underscored the scale of this threat, with over two-thirds of species detected, and nine of the ten most common species, threatened with extinction^4^. In response, CITES has expanded its listings to include 149 shark and ray species (Supplementary Material 1), such as the entire requiem shark family (Carcharhinidae, 54 species), which alone accounts for 68% of the global fin trade^3^. These listings require countries to issue Non-Detriment Findings (NDFs) and Legal Acquisition Findings (LAFs^5^) for all exports and imports, aiming to reduce opportunities for illegal trade.

Despite these measures, enforcing CITES regulations remains challenging. A primary obstacle is the difficulty of visually identifying dried fins, processed parts, or small fins from numerous species^6–8^. These products are easily disguised or mislabeled, allowing illegal shipments to pass undetected through porous borders and exposing countries to international sanctions^9^. Rapid in-port identification protocols have been developed and proven effective in some contexts^6,10–13^, but with the number of CITES-listed species continuing to grow, these tools must be re-evaluated for practicality, scalability, reproducibility, and cost-effectiveness. While species-specific primers are useful in targeted applications^6,10^, they become impractical for the broad, high-throughput identifications now required.

High-resolution melt (HRM) analysis represents a promising solution. HRM assays can be designed using universal primers for widely used barcoding loci (e.g., COI, cytB, 12S), coupled with intercalating dyes (e.g., SYTO9) that bind double-stranded DNA^14,15^. During heating, the dissociation of double-stranded DNA produces melt curve profiles that vary with nucleotide composition, generating unique species-specific signatures^16,17^. Moreover, advances in machine learning and image classification now enable the automated recognition of these profiles, making HRM not only sensitive and accurate but also suitable for rapid, high-throughput applications^18^.

Here we present a rapid (~ 2 h), cost-effective ($1.50 per sample), and broadly applicable assay for detecting elasmobranch species in trade. By generating distinctive curve profiles, we demonstrate that at least 55 species can be identified automatically using pretrained image classification models and a custom-built user-interface tool. This approach optimizes screening by reducing cost and time compared to sequencing or primer-based assays, while providing authorities with a scalable and reliable tool for real-time inspections of shipments suspected to contain illegally traded shark and ray products.

## Methodology

### Assay development and testing

To develop the universal assay, we used 669 vouchered samples—including alcohol-preserved tissue (560), dried fins (43), frozen meat (11), and genomic DNA extractions from large-scale processed fin market surveys (55)—representing 66 species (Table 1). Samples were sourced from collections at Florida International University, University of Florida, Mote Marine Laboratory, and the National Marine Fisheries Service. DNA was extracted using QuickExtract DNA Extraction Solution (Biosearch Technologies), a rapid, low-cost method suitable for field use. Briefly, a ~ 2 mm piece of tissue was placed in a PCR tube with 25 µL QuickExtract, incubated at room temperature for at least 10 min, then 15 µL of lysate was transferred to a MIC qPCR tube (Biomolecular Systems) and heated in a MIC thermal cycler at 65 °C for 6 min and 98 °C for 2 min, following manufacturer instructions. Lysates were used directly for PCR or stored at 4 °C short-term (up to a week) or − 20 °C long-term (up to six months).

**Table 1:** List of species and number of samples tested and included in the HRM curve profile database with their know geographic region (Western Atlantic [WAtl], Eastern Pacific [EPac], Indo-Pacific [IPac], Hong Kong [HK] markets, Indian Ocean [IOc], Western Pacific [WPac], Eastern Atlantic [EAtl], Central Pacific [CPac], North Atlantic [NAtl]).

Species	Common name	CITES	# Samples tested	Geographic origin
*Aetobatus narinari*	Spotted eagle ray		24	WAtl
*Alopias pelagicus*	Pelagic thresher shark	II	7	EPac
*Alopias superciliosus*	Bigeye thresher shark	II	4	EPac
*Alopias vulpinus*	Common thresher shark	II	9	EPac
*Carcharhinus acronotus*	Blacknose shark	II	7	WAtl
*Carcharhinus albimarginatus*	Silvertip shark	II	14	IPac
*Carcharhinus amblyrhynchos*	Grey reef shark	II	18	IPac
*Carcharhinus amboinensis*	Pigeye shark	II	10	HK markets
*Carcharhinus brachyurus*	Copper shark	II	16	IOc
*Carcharhinus brevipinna*	Spinner shark	II	10	WAtl
*Carcharhinus cerdale*	Pacific smalltail shark	II	4	EPac
*Carcharhinus dussumieri*	Whitecheek shark	II	8	HK markets
*Carcharhinus falciformis*	Silky shark	II	29	WAtl, EAtl, IOc, WPac, CPac, EPac,
*Carcharhinus fitzroyensis*	Creek shark	II	1	HK markets
*Carcharhinus isodon*	Finetooth shark	II	7	WAtl
*Carcharhinus leucas*	Bull shark	II	30	WAtl
*Carcharhinus limbatus*	Blacktip shark	II	30	WAtl
*Carcharhinus longimanus*	Oceanic whitetip shark	II	10	WAtl
*Carcharhinus melanopterus*	Blacktip reef shark	II	7	HK markets
*Carcharhinus obscurus*	Dusky shark	II	10	WAtl
*Carcharhinus perezi*	Caribbean reef shark	II	10	WAtl
*Carcharhinus plumbeus*	Sandbar shark	II	7	WAtl
*Carcharhinus porosus*	Smalltail shark	II	5	WAtl
*Carcharhinus signatus*	Night shark	II	12	WAtl
*Carcharhinus sorrah*	Spottail shark	II	6	HK markets
*Carcharias taurus*	Sand tiger shark		30	Unknown
*Carcharodon carcharias*	Great white shark	II	8	WAtl
*Centrophorus granulosus*	Gulper shark		7	HK markets
*Centroscymnus owstoni*	Roughskin dogfish		5	HK markets
*Chaenogaleus macrostoma*	Hooktooth shark		7	HK markets
*Chiloscyllium punctatum*	Brownbanded bamboo shark		8	HK markets
*Eusphyra blochii*	Winghead shark	II	2	HK markets
*Galeocerdo cuvier*	Tiger shark		10	WAtl
*Galeorhinus galeus*	School shark		10	HK markets
*Ginglymostoma cirratum*	Nurse shark		4	WAtl
*Glaucostegus cemiculus*	Blackchin guitarfish	II	8	HK markets
*Glaucostegus halavi*	Halavi guitarfish	II	8	HK markets
*Hemipristis elongata*	Snaggletooth shark		2	HK markets
*Hemitriakis japonica*	Japanese topeshark		7	HK markets
*Hypanus longus*	Longtail stingray		12	EPac
*Hypanus rubioi*	Longnose pacific stingray		8	EPac
*Isurus oxyrinchus*	Shortfin mako shark	II	7	WAtl
*Lamna nasus*	Portbeagle shark	II	13	NAtl
*Lamiopsis temminckii*	Broadfin shark	II	1	HK markets
*Mustelus antarcticus*	Gummy shark		7	HK markets
*Mustelus lunulatus*	Sicklefin smoothhound		1	HK markets
*Mustelus mosis*	Arabian smooth		6	HK markets
*Mustelus punctulatus*	Blackspotted smooth-hound		6	HK markets
*Mustelus schmitti*	Narrownose smooth-hound		7	HK markets
*Negaprion brevirostris*	Lemon shark	II	11	WAtl
*Prionace glauca*	Blue shark	II	34	WPac
*Pseudobatos planiceps*	Pacific guitarfish	II	6	EPac
*Raja clavata*	Thornback skate		8	NAtl
*Rhina ancylostoma*	Bowmouth guitarfish	II	7	Unknown
*Rhizoprionodon acutus*	Milk shark	II	14	HK markets
*Rhizoprionodon terraenovae*	Atlantic sharpnose shark	II	5	WAtl
*Sphyrna corona*	Scalloped bonnethead shark	II	15	EPac
*Sphyrna lewini*	Scalloped hammerhead	II	12	WAtl, EPac
*Sphyrna media*	Scoophead shark	II	2	EPac
*Sphyrna mokarran*	Great hammerhead	II	41	WAtl
Sphyrna tiburo*	Bonnethead shark	II	16	WAtl
*Sphyrna tudes*	Golden hammerhead	II	1	HK markets
*Sphyrna vespertina*	Pacific bonnethead shark	II	9	EPac
*Sphyrna zygaena*	Smooth hammerhead	II	6	Unknown
*Squalus cubensis*	Cuban dogfish		4	Unknown
*Stegostoma tigrinum*	Zebra shark		8	Unknown

*Sphyrna tiburo complex comprising *S. tiburo* and *S. alleni*.

Mitogenomes of 38 shark species spanning all Orders were downloaded from GenBank (Supplementary Material 2) to identify by eye a short region (100–250 bp) with high interspecific variation and conserved flanking sites for universal primer design. A region in the 12S rRNA gene, positions 766–1009 in the alignment, was selected. The forward primer (DNAid_Elasmo_F: 5′-ACGTCAGGTCGAGGTGTAG-3′) and reverse primer (DNAid_Elasmo_R: 5′-ATGTTACGACTTGCCTCCTCTT-3′) amplify a highly polymorphic 223 bp fragment.

Each 20 µL reaction contained 10 µL MeltDoctor HRM Master Mix (Applied Biosystems), 3.0 µL molecular-grade water, 2.5 µL of each primer (10 µM), and 2.0 µL extracted DNA. Cycling conditions were: 95 °C for 10 min; 40 cycles of 95 °C for 15 s, 56 °C for 30 s, and 72 °C for 30 s; final extension at 72 °C for 5 min; and a melt stage of 95 °C for 15 s, 60 °C for 60 s, then a ramp from 60 to 90 °C at 0.1 °C/s. All assay parameters can be uploaded directly into the MIC software using the Supplementary Material 3. Each run included a no-template control to assess reagent contamination. Amplicon sequences for all species tested are in GenBank (Accession: PV173532.1–PV173602.1).

**Fig. 1:**
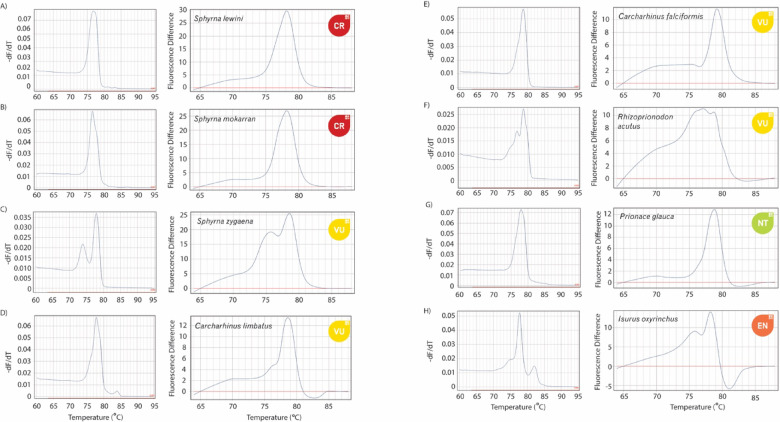
Comparison of melt (left) and derivative HRM (right) plots for eight (**A**-**G**) of the most common species in international markets. Colored symbols depict the IUCN Red List category for each species; Critically Endangered (CR), Endangered (EN), Vulnerable (VU), Near Threatened (NT). Melt curve (left) y-axis -dF/dT is the rate of change of fluorescence (F) with respect to temperature (T).

Initial trials showed that using the silky shark amplicon as a reference genotype produced the most distinctive HRM difference plots. Accordingly, a synthetic gBlock fragment of the silky shark target region (5 ng/µL; Supplementary Material 4) was used as a standardized positive control for fluorescence-difference plotting. This workflow yielded two distinctive profiles per sample, melt curves and derivative HRM plots (Fig. 1).

### Validation procedures

Assay specificity, sensitivity, and reproducibility were evaluated. In border-control contexts, shark and ray products typically include dried fins, carcasses, meat, or skin, all of which can be visually recognized as “shark/ray” by non-experts. This visual triage directs any assay user to elasmobranch products. However, given that a BLAST analysis showed a 100% primer match with non-elasmobranch taxa, specificity was further tested against six tuna and tuna-like species (Family Scombridae; *Katsuwonus pelamis, Thunnus obesus, T. orientalis, T. albacares, T. atlanticus*, and *A. rochei*), which could potentially be misidentified as shark meat in some scenarios.

Identification sensitivity was evaluated by determining the lowest normalized fluorescence signal at which species could still be correctly identified during assay development and testing. This threshold was expressed as the limit of identification (LOI), which we defined as the lowest signal level at which ≥ 95% of reactions produced correct species identifications. Reproducibility was assessed by asking an experienced scientist, uninvolved in assay development, to identify a blind set of ten ethanol-preserved fin-clip samples, each from a different species, using a user-interface tool (see below). Reproducibility was expressed as the percentage of samples correctly identified to species level.

### Performance with challenging samples

To evaluate performance on degraded material, two individual ceratotrichia from a single shark fin soup, and one unprocessed and one processed dried fins (> 4 years old) were cleaned with alcohol (70%) and processed using the same DNA extraction and qPCR protocols. Melt profiles were used for species identification, which was confirmed via endpoint PCR and sequencing following Cardeñosa et al. (2017).

### Species detection using image classification models

Melting curve images were generated using the Python library Matplotlib^19^, based on output files from MIC qPCR runs. Each image was saved as a formatted PNG file, with clearly labeled axes and legends. For every sample, three types of curves were produced: a standard melting curve, a HRM curve, and a derivative HRM curve (Supplementary Material 5). Each image was labeled with the corresponding species to enable supervised learning, a type of machine learning. To ensure adequate representation learning across classes (i.e., species), species with fewer than five samples were excluded, resulting in the removal of 30 samples from 12 species. The final dataset comprised 639 samples representing 55 species. This dataset was stratified by species and divided into training (60%), validation (20%), and test (20%) sets to maintain balanced class distributions. The training set was used to train image classification models, while the validation set was used to tune hyperparameters and select the optimal model. After identifying the best-performing hyperparameter configuration, models were retrained on the combined training and validation sets, considering them as a larger training set to maximize the volume of training data. The test set remained untouched throughout model development and was used solely for the final evaluation to ensure an unbiased performance assessment.

We tested a range of pretrained image classification models on the melting and HRM curve images, including traditional convolutional neural networks (i.e., ResNet^20^) and more advanced transformer-based architectures (i.e., Vision Transformer [ViT]^21^). All models were trained using the AdamW optimizer^22^. Hyperparameter tuning, including optimization of learning rate and weight decay, was performed via grid search. Each model was trained for 20 epochs per hyperparameter set, and validation accuracy was used to assess performance. Specifically, we recorded the highest validation accuracy achieved within each 20-epoch training cycle-referred to as the optimal validation accuracy. Final hyperparameters were selected based on the configuration that yielded the highest optimal validation accuracy. Models were then retrained for 20 epochs using the larger training dataset (training + validation sets), and their final performance was evaluated on the independent test set.

### Image classification experimental setting

Our experimental design focused on evaluating model performance, image input type, and the ability of models to handle previously unseen species. First, we compared three pretrained image classification models: ResNet18, ResNet50, and ViT. ResNet18 and ResNet50 contain 18 and 50 layers, respectively. Therefore, ResNet18 is a simpler model than ResNet50 with a smaller number of learnable parameters. For both ResNet architectures, we replaced the final fully connected layer to match the 55 species in our dataset and fine-tuned the models using our training data. ResNet50, with its deeper 50-layer structure compared to ResNet18’s 18 layers, allowed us to assess the impact of model depth on classification performance. The ViT model (google/vit-base-patch16-224), a transformer-based architecture, was similarly adapted and fine-tuned for the task. Each model was trained independently on three different types of melting curve images: again, standard melting curve, HRM curve, and derivative HRM curve. In addition to these individual inputs, we also evaluated a combined representation approach (i.e., using all the three melting curve images at once for species classification). For the combined input, we trained three independent instances of the same model architecture-one per image type-with no shared weights. Latent features were extracted from each model and concatenated, followed by a linear classification layer applied to the combined representation. Model performance was evaluated using standard classification metrics: accuracy, precision, recall, and F1-score^23^. Additionally, we recorded the average prediction confidence to assess model certainty in species prediction. To interpret model behavior, we analyzed feature attribution maps from the best-performing model to identify which image regions contributed most to the predictions. Misclassified samples were also reviewed to explore potential reasons for classification errors and better understand model limitations. Finally, we tested the models on samples from 12 species that were not included in the training data to test whether our model could determine whether given sample is not from predefined 55 species/classes. In other words, this was done to simulate real-world scenarios in which law enforcement personnel may encounter fins or body parts from species not listed under CITES or not previously validated. To evaluate the model’s performance on unseen species, we first identified a subset of unseen species from the whole dataset, consisting of 12 species with fewer than five samples each, totaling 30 samples. This subset is different from the training, validation, and test sets. We then used the best model to predict the unseen species. To further evaluate the model’s ability to recognize unseen species, we chose different confidence thresholds and merged the test set with the unseen species set (the original test set: 129 seen species samples and unseen species set: 30 unseen species samples, totaling 159 samples). Then, we had the model predict species within this combined dataset. If the prediction confidence was below the selected threshold, we labeled the sample as an “untested species”; otherwise, we classified it as a “tested species”. Finally, we assessed the model’s performance via precision, recall, and F1-score metrics.

## Results

### Assay developing and testing

**Fig. 2:**
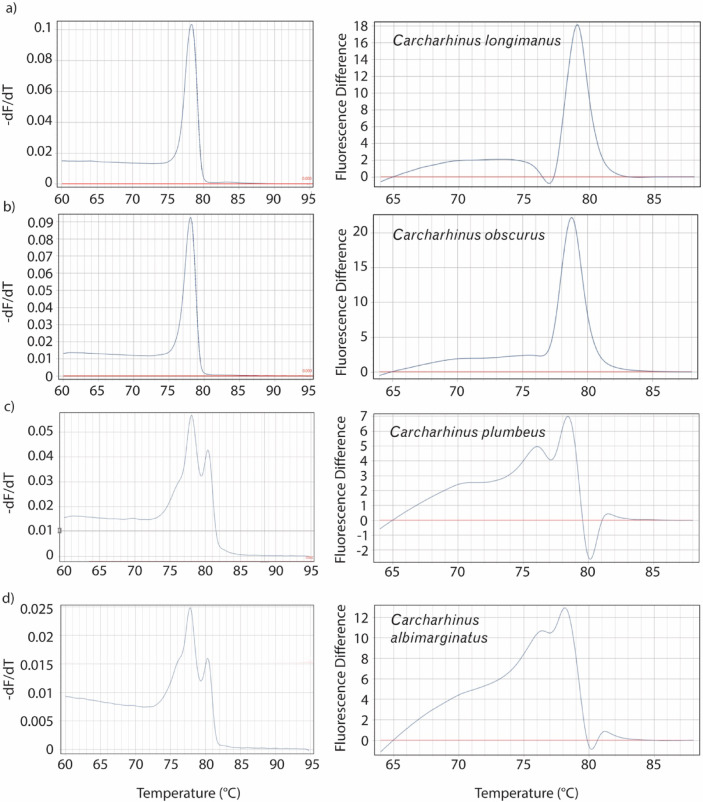
Melt (left) and HRM (right) plots for (**a**) oceanic whitetip shark (C. longimanus), (**b**) dusky shark (C. obscurus), (**c**) sandbar shark (C. plumbeus), and (**d**) silvertip shark (C. albimarginatus). Panels highlight the close similarity in melt profiles between species pairs (**a**–**b**) and (**c**–**d**), with small but detectable variations in their HRM profiles. In the melt curves (left), the y-axis represents –dF/dT, the negative rate of change in fluorescence (F) relative to temperature (T).

The field-based extraction method and the novel HRM assay designed for this study successfully amplified (i) frozen meat, (ii) alcohol-preserved, (iii) processed fins, (iv) dried fins, and (v) shark fin soup samples and yielded reproducible results to identify unknown samples. Most of the species tested yielded unique melt and derivative HRM curve profiles that were automatically identified with high accuracy (99.22%) by our image classification models (see below). However, in some instances, closely related species from the Carcharhinidae family yielded similar variants, almost identical melt curve profiles variants within 1° C (e.g., *C. longimanus* vs. *C. obscurus*; Fig. 2). These similarities are the result of only four nucleotide differences within the resulting amplicon (Supplementary Material 6). A test of five *C. obscurus* and *C. longimanus* samples using the custom-built user-interface tool (see below) resulted in correct species identification for 80% of the samples, with confidence values ranging from 0.99 to 0.74. The two misidentified samples included one *C. longimanus* classified as *C. obscurus* (confidence = 0.92) and one *C. obscurus* classified as *C. longimanus* (confidence = 0.91).

Of the 55 species tested, 38 (69%) were CITES listed species, with all of them generating identifiable curve profiles, different from the remaining 17 (31%) non-CITES listed species.

### Validation

All tuna samples produced amplification, melt, and derivative HRM curves (Supplementary Material 7), confirming that these primers amplify a broad range of taxa and are not specific to elasmobranchs. However, identification using the user-interface tool yielded low confidence values (0.36–0.08), producing no match with the reference curves of any target species.

**Fig. 3:**
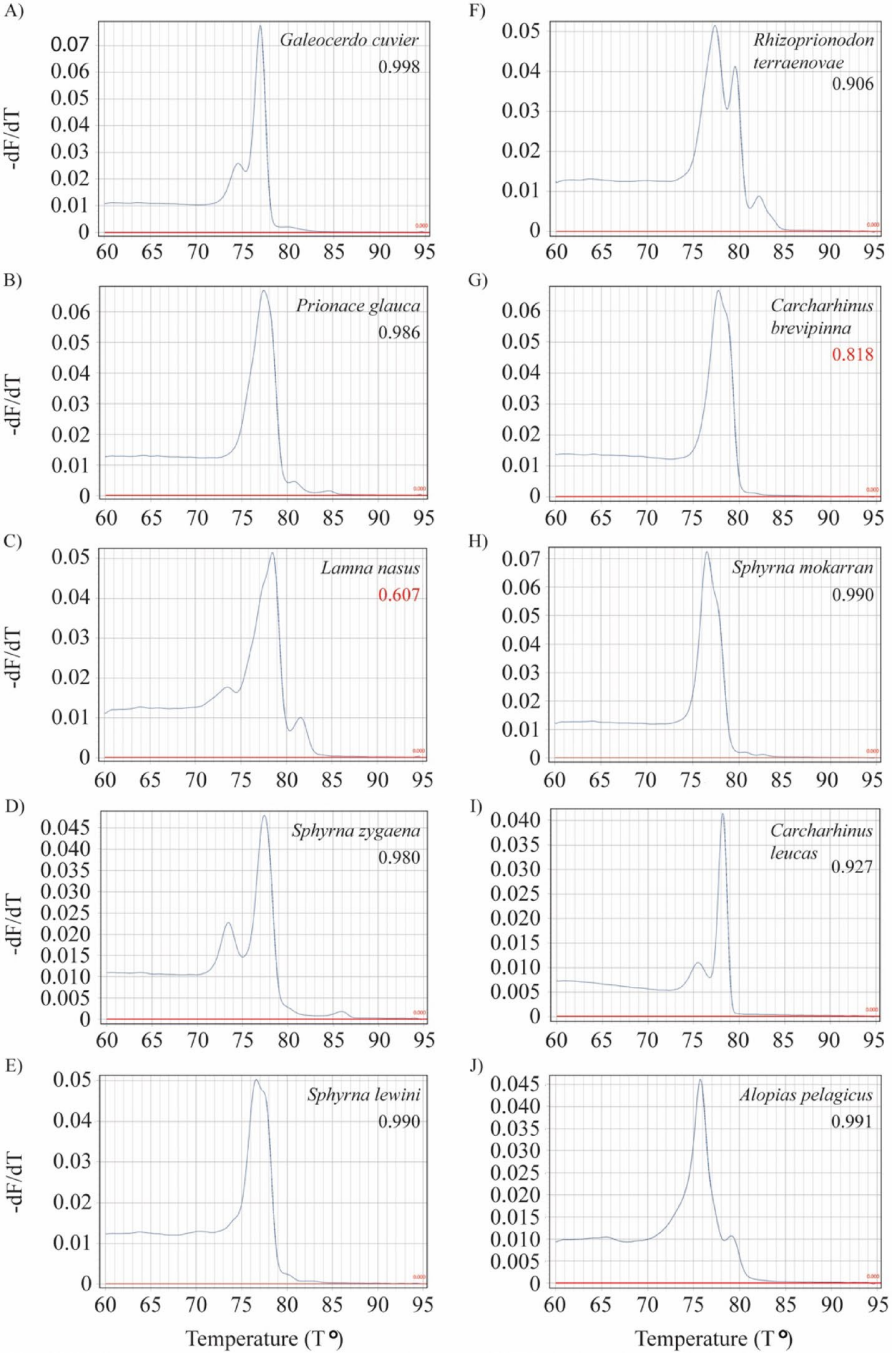
Melt curve profiles for ten species used for the blind test depicting the resulting confidence value for each profile. Red values represent values below the determined identification threshold of 0.90. The y-axis represents –dF/dT, the negative rate of change in fluorescence (F) relative to temperature (T).

The LOI was defined as the lowest normalized fluorescence signal at which ≥ 95% of reactions could still be correctly identified, which corresponded to a threshold of eight Normalized Fluorescence Units (NFUs). Moreover, during the testing phase, 16.5% of reactions did not reach this threshold, all of which produced melt and HRM curves that failed to match the expected species profiles and thus were not included in the profile database. Reproducibility was assessed by asking an experienced scientist, uninvolved in assay development, to identify a blind set of 10 ethanol-preserved fin-clip samples, each from a different species. All ten samples were correctly identified to the species level, yielding 100% reproducibility in terms of accuracy. Of these, eight returned confidence scores above the 0.90 threshold, while two were correctly identified but fell below this threshold (Fig. 3).

### Performance with challenging samples

All individual ceratotrichia from the bowl of shark fin soup, and the processed and unprocessed fins amplified correctly and produced curve profiles that were identified as silky and scalloped hammerhead sharks (soup) and as silky (processed) and blue shark (unprocessed) fins. The image classification model (see below) identified these samples with a confidence of 0.66, 0.89, 0.77, and 0.50 respectively. These identifications were also confirmed by sequencing.

### Image classification models

**Fig. 4:**
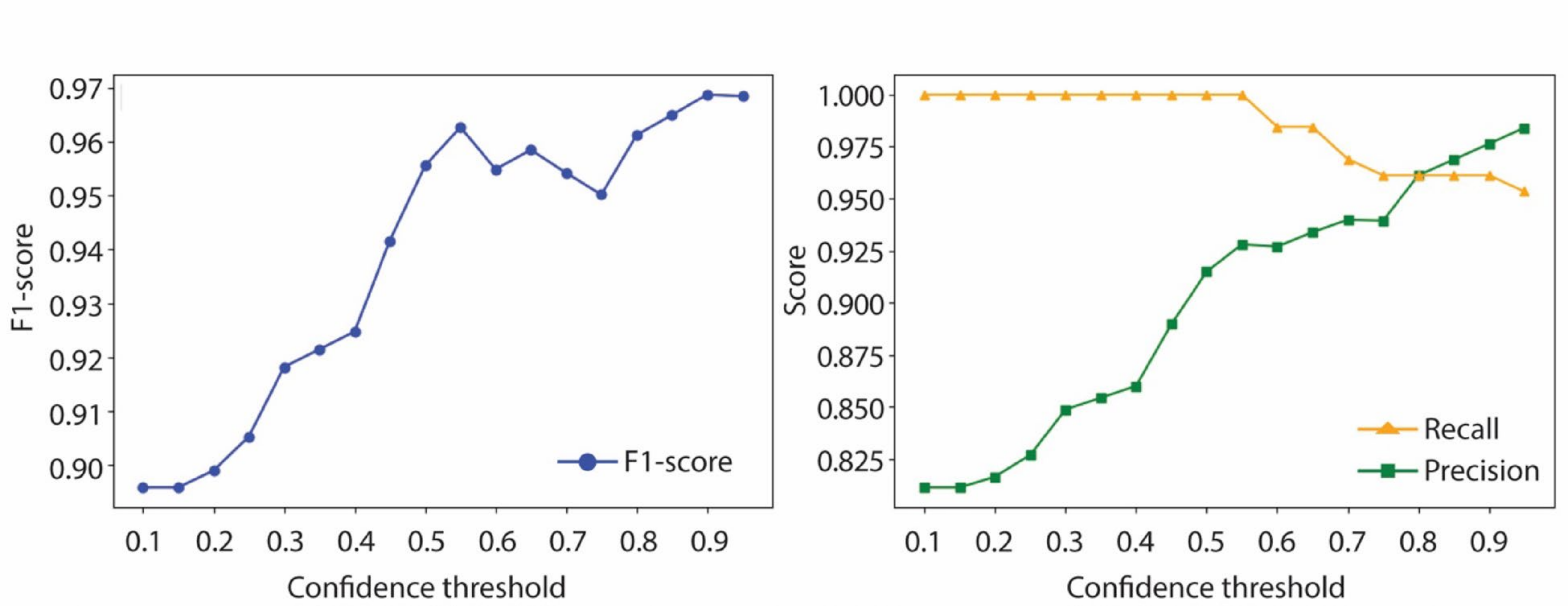
Performance of the model on unseen species. The model’s performance was evaluated using different confidence thresholds. The precision, recall, and F1-score are reported for each threshold.

Table 2 summarizes the classification performance across different model architectures and input types. Standard melting curve images consistently yielded the highest classification accuracy across all models. ResNet18 achieved the highest accuracy at 99.22%, followed by ResNet50 at 98.45%, both outperforming their counterparts trained on HRM or derivative HRM curve images. Combining all three image types—melting, HRM, and derivative HRM curves—did not improve classification accuracy but did increase the average prediction confidence (Table 2). A single misclassification case was observed, in which the model predicted *C. albimarginatus* instead of the true species *C. plumbeus* (Supplementary Material 8), two species known to produce highly similar melt profiles. When tested on previously unseen species, the model’s average prediction confidence dropped to 52.48%, reflecting lower certainty under unfamiliar conditions. The best performance for identifying such unknown samples was achieved using a confidence threshold of 0.9, suggesting that the model can effectively flag unrecognized species when an appropriate threshold is applied (Fig. 4).

**Table 2:** Overall performance of different models on different image types.

Model	Image Type	Accuracy	Precision	Recall	F1-Score	Avg. Confidence
ResNet18	Melting Curve	99.22%	0.9864	0.9922	0.9889	98.72%
	HRM Curve	98.45%	0.9826	0.9845	0.9812	98.42%
	Derivative HRM Curve	95.35%	0.9490	0.9535	0.9445	97.12%
	All three images	96.90%	0.9645	0.9690	0.9626	98.86%
ResNet50	Melting Curve	98.45%	0.9813	0.9845	0.9807	98.42%
	HRM Curve	97.67%	0.9787	0.9767	0.9734	98.41%
	Derivative HRM Curve	93.80%	0.9380	0.9380	0.9287	97.11%
	All three images	96.90%	0.9692	0.9690	0.9641	98.85%
ViT	Melting Curve	97.67%	0.9690	0.9767	0.9711	88.69%
	HRM Curve	95.35%	0.9554	0.9535	0.9494	87.37%
	Derivative HRM Curve	92.25%	0.9178	0.9225	0.9099	83.85%
	All three images	95.35%	0.9399	0.9535	0.9434	96.13%

Species identification from melt curves was supported by a custom-built user-interface tool (available at https://huggingface.co/spaces/luozhuanggary/HRM). This interface allows users to upload the qPCR run data (i.e., .csv file from the MIC software), visualize melt profiles, and obtain automated species assignments with confidence rates based on the reference curve library developed in this study.

## Discussion

Here we present a simple and cost-effective HRM assay capable of identifying 55 elasmobranch species, including 38 CITES-listed taxa, across a variety of sample types—fresh, frozen, processed, and cooked. The assay produced distinct melt and derivative HRM profiles allowing species identification using image classification models.

Over half of the tested species (36) displayed slight intraspecific variation in curve profiles, likely reflecting underlying genetic diversity or geographic population structure. However, because many samples were market-derived and lacked precise geographic origin, or came from single locations (e.g., Western Atlantic), this hypothesis could not be directly evaluated. The only species with confirmed origins spanning a broad geographic range (i.e., *C. falciformis*; Eastern Pacific, Central Pacific, Western Pacific, Indian Ocean, Eastern Atlantic, and Western Atlantic) showed no variation in melt or HRM profiles that would compromise accurate species identification (Supplementary Material 9). This consistency is expected given that the 12S is a coding, highly conserved, mitochondrial locus with a low mutation rate and limited nucleotide differentiation at the population level^24^. However, future work should expand the reference library with geographically verified samples is needed to fully evaluate potential population-level variations.

Beyond locus-level stability, our validation experiments further demonstrated the robustness of the assay. The limit of identification (LOI) was set conservatively at eight RFUs, below which reactions failed to yield reliable species-level matches, ensuring that low-quality signals are excluded.

Although the primers amplified non-elasmobranch DNA (e.g., tuna species), these reactions consistently produced low confidence values (0.36–0.08), preventing misidentification when non-elasmobranch species are present. Reproducibility was high, with all blind samples correctly identified to species level. While two samples produced confidence values below the 0.90 threshold, they were still accurately classified, underscoring that the threshold acts as a conservative safeguard to flag uncertain cases rather than a failure of the method. Importantly, even highly processed samples, such as individual ceratotrichia from shark fin soup typically cooked alongside proteins from other taxa, and old processed and unprocessed fins, produced identifiable profiles that matched silky, scalloped hammerhead, and blue sharks. In these challenging cases, the image classification model returned correct identifications but with relatively low confidence values (< 0.9), likely reflecting (i) matrix effects from mixed-source DNA, (ii) reduced signal quality near the LOI due to the degraded nature of these types of samples^25^, and (iii) limited representation of these sample types in the training database. These outcomes suggest that while the assay is capable of handling degraded samples, expanding the reference library with additional replicates and diverse matrices will further increase confidence for difficult samples in the future. Together, these results underscore that the assay not only achieves high accuracy but also incorporates built-in safeguards—confidence thresholds and reference-curve validation—that minimize the risk of false positives.

Close similarities in melting and HRM curve profiles were observed between the oceanic whitetip (*C. longimanus*) and dusky shark (*C. obscurus*), as well as between the sandbar shark (*C. plumbeus*) and silvertip shark (*C. albimarginatus*; Fig. 2). These findings are consistent with previous reports highlighting the challenge of distinguishing certain species within the *Carcharhinus* genus^26^. At the sequence level, four nucleotide differences were identified between dusky and oceanic whitetip sharks within the target amplicon, while sandbar and silvertip sharks differed by 12 nucleotides (Supplementary Material 6). Although Galapagos sharks (*C. galapagensis*) were not included in our tests, comparisons of GenBank sequences (Accession numbers: NC020611.1 – OR722519.1) suggest that their melt profiles would likely resemble those of *C. obscurus*, given the single-nucleotide difference observed in the target region. Despite the close profile similarities among these species, our image classification models achieved high accuracy in distinguishing them (see below). For example, one misclassification occurred in which *C. albimarginatus* was predicted instead of the true species *C. plumbeus*. This error likely stemmed from the high degree of similarity between their melting curves. Furthermore, a test with five *C. obscurus* and five *C. longimanus* using the custom-built user-interface tool (https://huggingface.co/spaces/luozhuanggary/HRM) resulted in correct identification for 80% of the samples. Misidentifications included one *C. longimanus* classified as *C. obscurus* (confidence = 0.92) and one *C. obscurus* classified as *C. longimanus* (confidence = 0.91), reflecting the close genetic relationship between these species. In cases where definitive species-level resolution among *C. obscurus*, *C. longimanus*, and potentially *C. galapagensis* is required, sequencing is recommended. However, in most law enforcement contexts, this level of resolution is not necessary. Because the primary application of this tool is to detect illicit trade during border control inspection, any result indicating *C. longimanus/C. obscurus* in consignments lacking CITES permits, or with permits listing different species, would provide sufficient grounds for authorities to initiate a detailed inspection and, if needed, confirmatory Sanger sequencing.

**Fig. 5:**
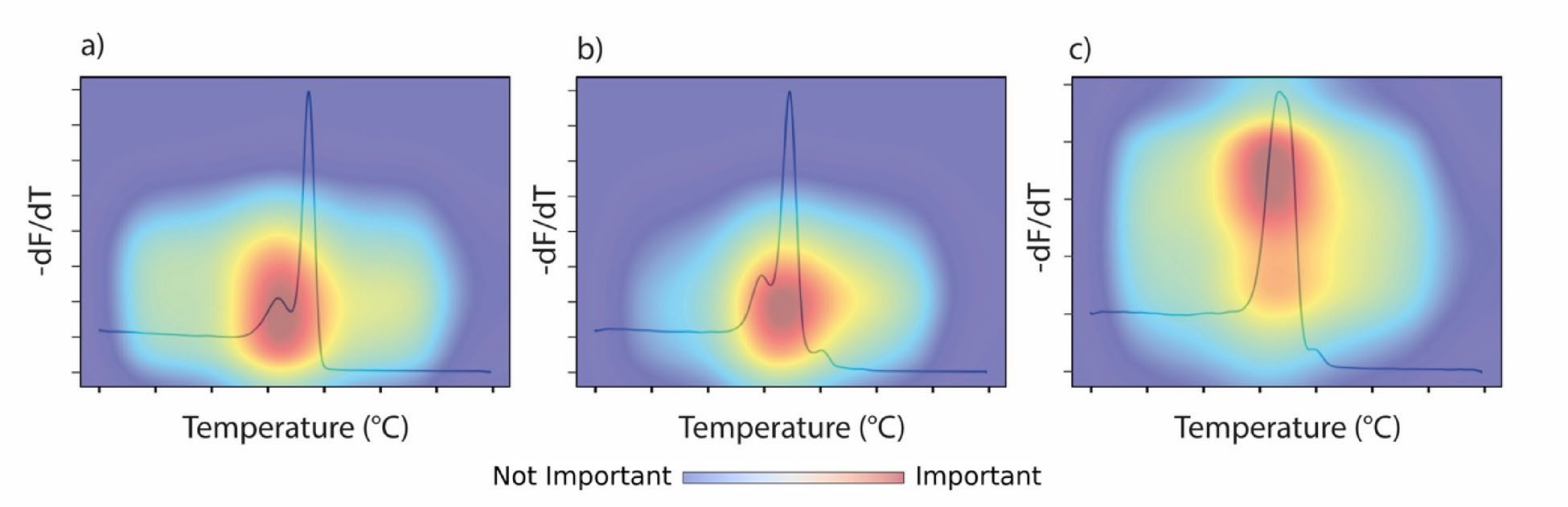
Grad-CAM heatmaps for the best model (ReNet18) on melting curve images. The heatmaps highlight the most informative/important regions of the melting curve that contribute to the model’s prediction for three different species (**a**) Bull shark (*Carcharhinus leucas*), (**b**) tiger shark (*Galeocerdo cuvier*), and (**c**) scalloped hammerhead (*Sphyrna lewini*). Melt curve y-axis -dF/dT is the rate of change of fluorescence (F) with respect to temperature (T).

In our image classification experiments, ResNet18, despite its simplicity, achieved the highest classification accuracy (99.22%) when trained on melting curve images, outperforming deeper models like ResNet50 (98.45%) and the transformer-based ViT (97.67%). This suggests that the dataset size and complexity favor lightweight architectures. Combining all three image types (melting, HRM, derivative HRM curve images) did not improve accuracy but boosted average confidence (e.g., ViT improved from 88.69% to 96.13%), indicating complementary value across representations while highlighting that the standard melting curve provides the most informative signal for species-level classification. Future work could explore adaptive fusion or attention-based mechanisms^27^ to further leverage these combined inputs. Grad-CAM^28^ visualizations from ResNet18 confirmed that the model focused on key curve regions, supporting its interpretability (Fig. 5). The model’s average prediction confidence dropped to 52.48% when applied to untested species, indicating its utility for flagging potentially unknown or misidentified samples. The optimal threshold for detecting unknowns was 0.9, offering a practical tool for enforcement scenarios.

Previous field-based identification protocols relied on species-specific primers, which are useful when dealing with a limited number of species^6,13,29,30^. However, these methods face significant challenges when it comes to identifying many closely related species (e.g., family Carcharhinidae). While species-specific primers can be multiplexed^6^, the number of primers that can be used together is limited by the need to maintain consistent conditions across the multiplex, avoid primer interactions, and ensure PCR efficiency is not compromised^31^. Moreover, inspections by law enforcement of elasmobranch products comprise many tons and tens of thousands of samples (e.g., shark fins)^7,32^. Even if several multiplexes existed to identify many species, there would have to be a compromise in the number of samples to be detected, or the time of screening a large-volume consignment. Our universal assay overcomes these limitations with its low cost (~ $1.50 USD/sample), rapid turnaround (~ 2 h), and broad applicability to degraded and diverse sample types (Table 3). As visually identifying small or processed fins is increasingly difficult, particularly in regions where early processing is common^7^, molecular tools are essential to ensure compliance with CITES and detect undeclared species.

**Table 3:** A comparison of available quick, field-based elasmobranch identification tools.

Reference	# of species	Chemistry	Cost per sample (USD)	Time to identification of 1–10 samples	Time to identification of > 45 samples
This study	At least 67	qPCR—HRM^a^	$1.50	~ 2 h	~ 2 h
Cardenosa et al.^6^	9	qPCR—ssp^b^	$0.94	~ 3 h	~ 3 h
Prasetyo et al ^11^	At least 28	qPCR—probes^c^	~ $15.00	~ 2.5 h	~ 2.5 h
But et al.^30^	12	LAMP^d^	$0.60	~ 3 h	~ 14 h
Tiktak et al.^13^	3	LAMP^d^	$6.00	?	?

^a^ Quantitative PCR High Resolution Melt, ^b^ Quantitative PCR with species specific primers, ^c^ Quantitative PCR with hybridizing probes (exact chemistry of this assay is not disclosed), ^d^ Loop-mediated isothermal amplification assay. ? denotes that total times are not explicitly mentioned in the original study.

With many of the most highly traded elasmobranch species now listed under CITES Appendix II, virtually all consignments should be presented at international borders with NDF and LAF certificates. While these certificates may cover one or several species (e.g., blue shark [*Prionace glauca*], milk shark [*Rhizoprionodon acutus*]), they do not necessarily reflect the entire species composition of the consignment. To ensure compliance with CITES requirements and accurate species declarations, visual identification guides, and our assay can be utilized whenever possible. However, visual identification is not always feasible, particularly with small fins^3^, and processed products like salted meat, processed shark fins, and dried skins. In fact, the early processing of fins is becoming more common in some South and Central American countries, possibly to evade visual identification of regulated species^7^. In such cases, molecular identification tools, capable of detecting large volumes of highly processed products, are crucial for identifying species and combating illegal trade.

The HRM assay presented here is, to our knowledge, the most comprehensive species-level identification tool of its kind, capable of detecting the greatest number of elasmobranch species using high-resolution melt analysis to date. As regulatory frameworks expand to include more protected taxa, the need for scalable, rapid, and field-deployable genetic tools becomes increasingly urgent. Future efforts should aim to broaden the species coverage of HRM assays and evaluate their limits in resolving taxonomic complexity across diverse lineages. Beyond elasmobranchs, this approach offers a scalable blueprint for applying molecular surveillance across multiple taxonomic groups involved in the global wildlife trade. By enabling rapid, on-site identification of trafficked species during inspections, tools like this HRM assay could become cornerstones in the international fight against illegal wildlife trafficking and a critical component of real-time enforcement at trade hotspots worldwide.

## Supplementary Information

Below is the link to the electronic supplementary material.

Supplementary Material 1

Supplementary Material 2

Supplementary Material 3

Supplementary Material 4

Supplementary Material 5

Supplementary Material 6

Supplementary Material 7

Supplementary Material 8

Supplementary Material 9

## Data Availability

All data are available in the main text or the supplementary materials. Amplicon sequences for all species tested can be found in GenBank (Accession: PV173532.1 – PV173602.1). Computer code for model can be found at https://web.cs.wpi.edu/ ~ kmlee/HRM.zip.
