# Supplementary material for: Integrating portable qPCR and image recognition to combat illegal trade in sharks and rays
Source: Sci Rep. 2025 Nov 4;15:38629. doi: 10.1038/s41598-025-22370-y (PMC12586682; doi:10.1038/s41598-025-22370-y)
Supplement: Supplementary file 1 — Supplementary Material 1 [file 41598_2025_22370_MOESM1_ESM.docx]

Table 1. List of elasmobranch species listed on CITES Appendices from 2003 to 2022 showing their IUCN Red List status (if assessed), their CITES listing Appendix, the Conference of the Parties (CoP) where listings occurred, and the year where listings took effect.

| Species | Common name | IUCN | Appendix | CoP | Effective year |
| --- | --- | --- | --- | --- | --- |
| *Cetorhinus maximus* | Basking shark | EN | II | CoP12 | 2003 |
| *Rhincodon typus* | Whale shark | EN | II | CoP12 | 2003 |
| *Carcharodon carcharias* | Great white shark | VU | II | CoP13 | 2005 |
| *Pristis pristis* | Largetooth sawfish | CR | I | CoP14 | 2007 |
| *Pristis zijsron* | Green sawfish | CR | I | CoP14 | 2007 |
| *Pristis pectinate* | Smalltooth sawfish | CR | I | CoP14 | 2007 |
| *Pristis clavate* | Dwarf sawfish | CR | I | CoP14 | 2007 |
| *Anoxypristis cuspidata* | Narrow sawfish | EN | I | CoP14 | 2007 |
| *Carcharhinus longimanus* | Oceanic whitetip shark | CR | II | CoP16 | 2014 |
| *Sphyrna lewini* | Scalloped hammerhead | CR | II | CoP16 | 2014 |
| *Sphyrna zygaena* | Smooth hammerhead | VU | II | CoP16 | 2014 |
| *Sphyrna mokarran* | Great hammerhead | CR | II | CoP16 | 2014 |
| *Lamna nasus* | Porbeagle shark | VU | II | CoP16 | 2014 |
| *Mobula birostris* | Oceanic manta ray | EN | II | CoP16 | 2014 |
| *Mobula alfredi* | Reef manta ray | VU | II | CoP16 | 2014 |
| *Mobula eregoodoo* | Pigmy devil ray | EN | II | CoP17 | 2017 |
| *Mobula hypostoma* | Lesser devil ray | EN | II | CoP17 | 2017 |
| *Mobula kuhlii* | Shortfin devil ray | EN | II | CoP17 | 2017 |
| *Mobula mobular* | Devil ray | EN | II | CoP17 | 2017 |
| *Mobula munkiana* | Munk’s devil ray | EN | II | CoP17 | 2017 |
| *Mobula tarapacana* | Chilean devil ray | EN | II | CoP17 | 2017 |
| *Mobula thurstoni* | Bentfin devil ray | EN | II | CoP17 | 2017 |
| *Alopias pelagicus* | Pelagic thresher shark | EN | II | CoP17 | 2017 |
| *Alopias superciliosus* | Bigeye thresher shark | VU | II | CoP17 | 2017 |
| *Alopias vulpinus* | Common thresher shark | VU | II | CoP17 | 2017 |
| *Carcharhinus falciformis* | Silky shark | VU | II | CoP17 | 2017 |
| *Isurus oxyrinchus* | Shortfin mako shark | EN | II | CoP18 | 2019 |
| *Isurus paucus* | Longfin mako shark | EN | II | CoP18 | 2019 |
| *Glaucostegus halavi* | Halavi guitarfish | CR | II | CoP18 | 2019 |
| *Glaucostegus cemiculus* | Blackchin guitarfish | CR | II | CoP18 | 2019 |
| *Glaucostegus thouin* | Clubnose guitarfish | CR | II | CoP18 | 2019 |
| *Glaucostegus younholeei* | Bangladeshi guitarfish | CR | II | CoP18 | 2019 |
| *Glaucostegus obtusus* | Widenose guitarfish | CR | II | CoP18 | 2019 |
| *Glaucostegus typus* | Giant guitarfish | CR | II | CoP18 | 2019 |
| *Glaucostegus granulatus* | Sharpnose guitarfish | CR | II | CoP18 | 2019 |
| *Rhina ancylostoma* | Bowmouth guitarfish | CR | II | CoP18 | 2019 |
| *Rhynchobatus australiae* | Bottlenose wedgefish | CR | II | CoP18 | 2019 |
| *Rhynchobatus cooki* | Clown wedgefish | CR | II | CoP18 | 2019 |
| *Rhynchobatus djiddensis* | White-spotted wedgefish | CR | II | CoP18 | 2019 |
| *Rhynchobatus immaculatus* | Taiwanese wedgefish | CR | II | CoP18 | 2019 |
| *Rhynchobatus laevis* | Smoothnose wedgefish | CR | II | CoP18 | 2019 |
| *Rhynchobatus springeri* | Broadnose wedgefish | CR | II | CoP18 | 2019 |
| *Rhynchobatus palpebratus* | Eyebrow wedgefish | NT | II | CoP18 | 2019 |
| *Rhynchobatus luebberti* | African wedgefish | CR | II | CoP18 | 2019 |
| *Carcharhinus acronotus* | Blacknose shark | EN | II | CoP19 | 2023 |
| *Carcharhinus albimarginatus* | Silvertip shark | VU | II | CoP19 | 2023 |
| *Carcharhinus altimus* | Bignose shark | EN | II | CoP19 | 2023 |
| *Carcharhinus amblyrhynchoides* | Graceful shark | VU | II | CoP19 | 2023 |
| *Carcharhinus amblyrhynchos* | Grey reef shark | EN | II | CoP19 | 2023 |
| *Carcharhinus amboinensis* | Pigeye shark | VU | II | CoP19 | 2023 |
| *Carcharhinus borneensis* | Borneo shark | CR | II | CoP19 | 2023 |
| *Carcharhinus brachyurus* | Bronze whaler | VU | II | CoP19 | 2023 |
| *Carcharhinus brevipinna* | Spinner shark | VU | II | CoP19 | 2023 |
| *Carcharhinus cautus* | Nervous shark | LC | II | CoP19 | 2023 |
| *Carcharhinus cerdale* | Pacific smalltail shark | LC | II | CoP19 | 2023 |
| *Carcharhinus coatesi* | Coates’s shark | LC | II | CoP19 | 2023 |
| *Carcharhinus dussumieri* | Whitecheek shark | EN | II | CoP19 | 2023 |
| *Carcharhinus fitzroyensis* | Creek shark | LC | II | CoP19 | 2023 |
| *Carcharhinus galapagensis* | Galapagos sharks | LC | II | CoP19 | 2023 |
| *Carcharhinus hemiodon* | Pondicherry shark | CR | II | CoP19 | 2023 |
| *Carcharhinus humani* | Human’s whaler shark | DD | II | CoP19 | 2023 |
| *Carcharhinus melanopterus* | Blacktip reef shark | VU | II | CoP19 | 2023 |
| *Carcharhinus limbatus* | Blacktip shark | VU | II | CoP19 | 2023 |
| *Carcharhinus leucas* | Bull shark | VU | II | CoP19 | 2023 |
| *Carcharhinus perezi* | Caribbean reef shark | EN | II | CoP19 | 2023 |
| *Carcharhinus plumbeus* | Sandbar shark | EN | II | CoP19 | 2023 |
| *Carcharhinus porosus* | Smalltail shark | CR | II | CoP19 | 2023 |
| *Carcharhinus macloti* | Hardnose shark | NT | II | CoP19 | 2023 |
| *Carcharhinus tjutjot* | Indonesian whaler shark | VU | II | CoP19 | 2023 |
| *Carcharhinus signatus* | Night shark | EN | II | CoP19 | 2023 |
| *Carcharhinus tilstoni* | Australian blacktip shark | LC | II | CoP19 | 2023 |
| *Carcharhinus sorrah* | Spottail shark | NT | II | CoP19 | 2023 |
| *Carcharhinus coatesi* | Australian blackspot shark | LC | II | CoP19 | 2023 |
| *Carcharhinus obscurus* | Dusky shark | EN | II | CoP19 | 2023 |
| *Carcharhinus leiodon* | Smoothtooth blacktip | EN | II | CoP19 | 2023 |
| *Carcharhinus sealei* | Blackspot shark | VU | II | CoP19 | 2023 |
| *Carcharhinus isodon* | Finetooth shark | NT | II | CoP19 | 2023 |
| *Carcharhinus obsoletus* | Lost shark | CR | II | CoP19 | 2023 |
| *Lamiopsis tephrodes* | Borneo broadfin shark | EN | II | CoP19 | 2023 |
| *Nasolamia velox* | Whitenose shark | EN | II | CoP19 | 2023 |
| *Lamiopsis temminckii* | Broadfin shark | EN | II | CoP19 | 2023 |
| *Rhizoprionodon acutus* | Milk shark | VU | II | CoP19 | 2023 |
| *Rhizoprionodon oligolinx* | Grey sharpnose shark | NT | II | CoP19 | 2023 |
| *Rhizoprionodon terraenovae* | Atlantic sharpnose shark | LC | II | CoP19 | 2023 |
| *Rhizoprionodon longurio* | Pacific sharpnose shark | VU | II | CoP19 | 2023 |
| *Rhizoprionodon lalandii* | Brazilian sharpnose shark | VU | II | CoP19 | 2023 |
| *Rhizoprionodon porosus* | Caribbean sharpnose shark | VU | II | CoP19 | 2023 |
| *Rhizoprionodon taylori* | Australian sharpnoseshark | LC | II | CoP19 | 2023 |
| *Scoliodon laticaudus* | Spadenose shark | NT | II | CoP19 | 2023 |
| *Scoliodon macrorhynchos* | Pacific spadenose shark | NT | II | CoP19 | 2023 |
| *Glyphis gangeticus* | Ganges shark | CR | II | CoP19 | 2023 |
| *Glyphis garricki* | Northern river shark | VU | II | CoP19 | 2023 |
| *Glyphis glyphis* | Speartooth shark | VU | II | CoP19 | 2023 |
| *Negaprion brevirostris* | Lemon shark | VU | II | CoP19 | 2023 |
| *Negaprion acutidens* | Sharptooth lemon shark | VU | II | CoP19 | 2023 |
| *Prionace glauca* | Blue shark | NT | II | CoP19 | 2023 |
| *Loxodon macrorhinus* | Sliteye shark | NT | II | CoP19 | 2023 |
| *Isogomphodon oxyrhynchus* | Daggernose shark | CR | II | CoP19 | 2023 |
| *Triaenodon obesus* | Whitetip reef shark | VU | II | CoP19 | 2023 |
| *Sphyrna corona* | Scalloped bonnethead shark | CR | II | CoP19 | 2023 |
| *Sphyrna tiburo* | Bonnethead shark | EN | II | CoP19 | 2023 |
| *Sphyrna media* | Scoophead shark | CR | II | CoP19 | 2023 |
| *Sphyrna tudes* | Smalleye hammerhead | CR | II | CoP19 | 2023 |
| *Eusphyra blochii* | Winghead shark | EN | II | CoP19 | 2023 |
| *Sphyrna gilberti* | Carolina hammerhead | DD | II | CoP19 | 2023 |
| *Potamotrygon albimaculata* | Black river stingray |  | II | CoP19 | 2023 |
| *Potamotrygon henlei* | Bigtooth river stingray | LC | II | CoP19 | 2023 |
| *Potamotrygon jabuti* | Pearl freshwater stingray |  | II | CoP19 | 2023 |
| *Potamotrygon leopoldi* | Xingu freshwater stingray | VU | II | CoP19 | 2023 |
| *Potamotrygon marquesi* | Ocellate river stingray |  | II | CoP19 | 2023 |
| *Potamotrygon signata* | Parnaiba river stingray | DD | II | CoP19 | 2023 |
| *Potamotrygon wallacei* | Porcupine stringray |  | II | CoP19 | 2023 |
| *Acroteriobatus variegatus* | Stripenose guitarfish | CR | II | CoP19 | 2023 |
| *Acroteriobatus andysabini* | Malagasy blue-spotted guitarfish |  | II | CoP19 | 2023 |
| *Acroteriobatus annulatus* | Lesser guitarfish | VU | II | CoP19 | 2023 |
| *Acroteriobatus blochii* | Bluntnose guitarfish | LC | II | CoP19 | 2023 |
| *Acroteriobatus leucospilus* | Greyspotted guitarfish | EN | II | CoP19 | 2023 |
| *Acroteriobatus acellatus* | Speckled guitarfish | DD | II | CoP19 | 2023 |
| *Acroteriobatus omanensis* | Oman guitarfish | DD | II | CoP19 | 2023 |
| *Acroteriobatus salalah* | Salalah guitarfish | NT | II | CoP19 | 2023 |
| *Acroteriobatus stehmanni* | Socotra blue-spotted guitarfish |  | II | CoP19 | 2023 |
| *Acroteriobatus zanzibarensis* | Zanzibar guitarfish | NT | II | CoP19 | 2023 |
| *Pseudobatos horkelii* | Brazilian guitarfish | CR | II | CoP19 | 2023 |
| *Pseudobatos buthi* | Spadenose guitarfish | VU | II | CoP19 | 2023 |
| *Pseudobatos glaucostigma* | Speckled guitarfish | VU | II | CoP19 | 2023 |
| *Pseudobatos lentiginosus* | Atlantic guitarfish | VU | II | CoP19 | 2023 |
| *Pseudobatos leucorhynchus* | Whitesnout guitarfish | VU | II | CoP19 | 2023 |
| *Pseudobatos percellens* | Chola guitarfish | EN | II | CoP19 | 2023 |
| *Pseudobatos planiceps* | Pacific guitarfish | VU | II | CoP19 | 2023 |
| *Pseudobatos prahlii* | Gorgona guitarfish | VU | II | CoP19 | 2023 |
| *Pseudobatos productus* | Shovelnose guitarfish | NT | II | CoP19 | 2023 |
| *Rhinobatos albomaculatus* | Whitespotted guitarfish | CR | II | CoP19 | 2023 |
| *Rhinobatos annandalei* | Annandale’s guitarfish | CR | II | CoP19 | 2023 |
| *Rhinobatos austini* | Austin’s guitarfish | DD | II | CoP19 | 2023 |
| *Rhinobatos borneensis* | Borneo guitarfish | EN | II | CoP19 | 2023 |
| *Rhinobatos holcorhynchus* | Slender guitarfish | DD | II | CoP19 | 2023 |
| *Rhinobatos hynnicephalus* | Ringed guitarfish | EN | II | CoP19 | 2023 |
| *Rhinobatos irvinei* | Spineback guitarfish | CR | II | CoP19 | 2023 |
| *Rhinobatos jimbaranensis* | Jimbaran shovelnose ray | CR | II | CoP19 | 2023 |
| *Rhinobatos lionotus* | Smoothback guitarfish | CR | II | CoP19 | 2023 |
| *Rhinobatos mania* | Papuan guitarfish | LC | II | CoP19 | 2023 |
| *Rhinobatos nudidorsalis* | Bareback guitarfish | DD | II | CoP19 | 2023 |
| *Rhinobatos penggali* | Indonesian shovelnose ray | EN | II | CoP19 | 2023 |
| *Rhinobatos punctifer* | Spotted guitarfish | NT | II | CoP19 | 2023 |
| *Rhinobatos ranongensis* | Ranong guitarfish | VU | II | CoP19 | 2023 |
| *Rhinobatos rhinobatos* | Common guitarfish | CR | II | CoP19 | 2023 |
| *Rhinobatos sainsburyi* | Goldeneye shovelnose ray | VU | II | CoP19 | 2023 |
| *Rhinobatos schlegelii* | Brown guitarfish | CR | II | CoP19 | 2023 |
| *Rhinobatos whitei* | Philippine guitarfish | CR | II | CoP19 | 2023 |
