# Supplementary figures and images for: Integrating portable qPCR and image recognition to combat illegal trade in sharks and rays
Source: Sci Rep. 2025 Nov 4;15:38629. doi: 10.1038/s41598-025-22370-y (PMC12586682; doi:10.1038/s41598-025-22370-y)

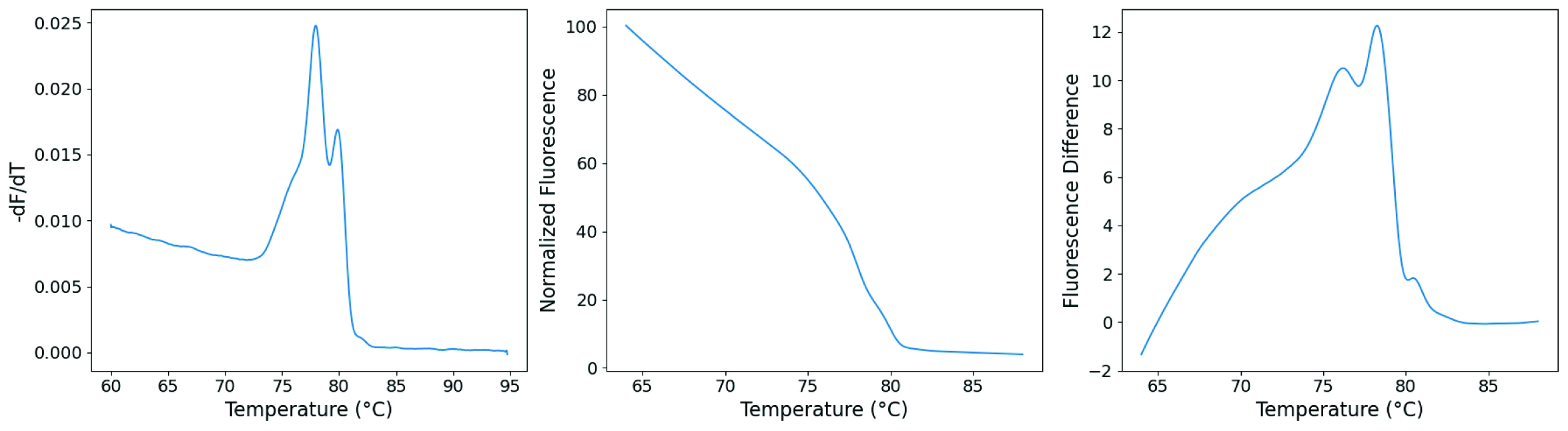

Supplement: Supplementary file 5 — Supplementary Material 5 [file 41598_2025_22370_MOESM5_ESM.jpg]

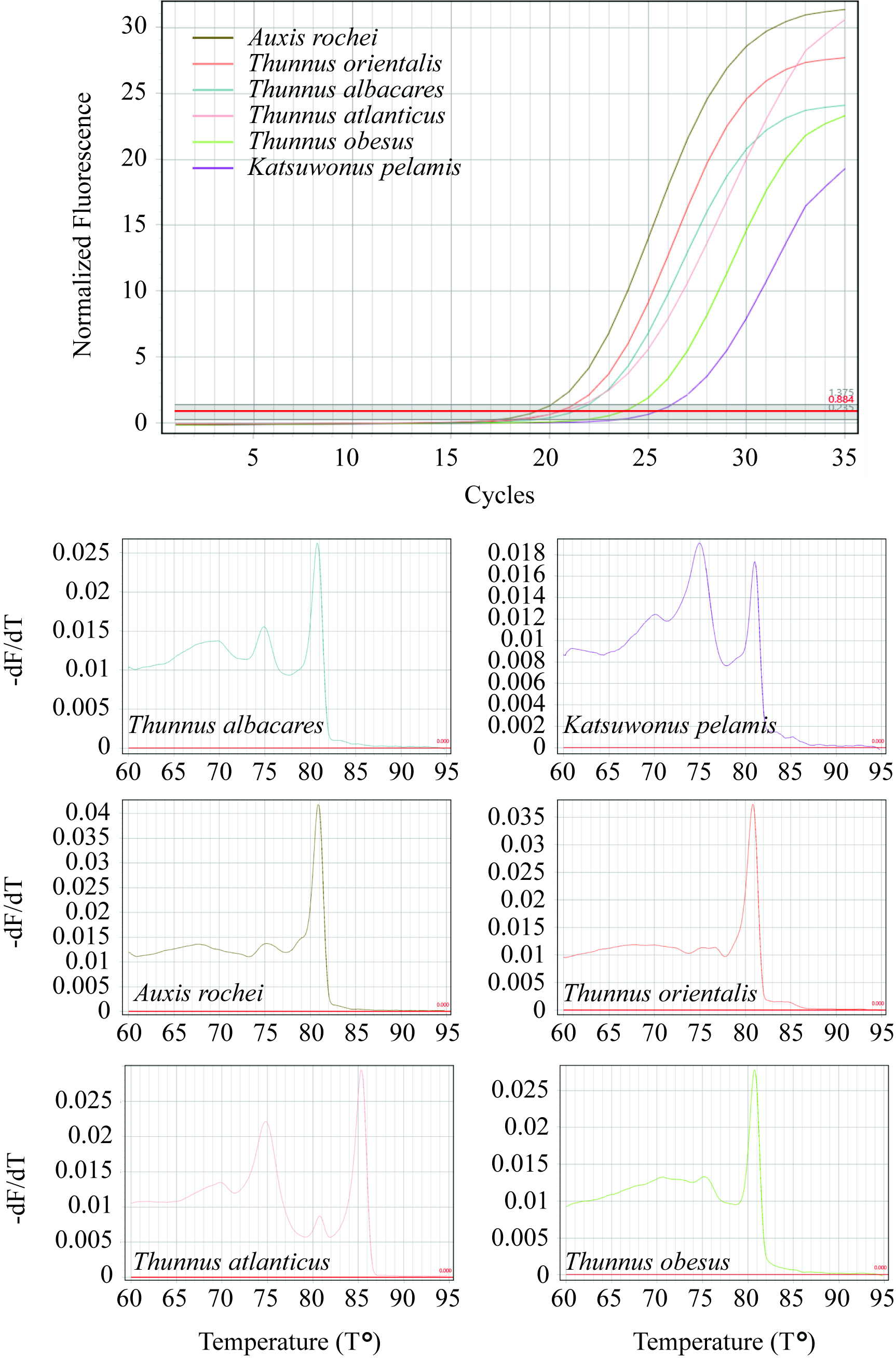

Supplement: Supplementary file 7 — Supplementary Material 7 [file 41598_2025_22370_MOESM7_ESM.jpg]

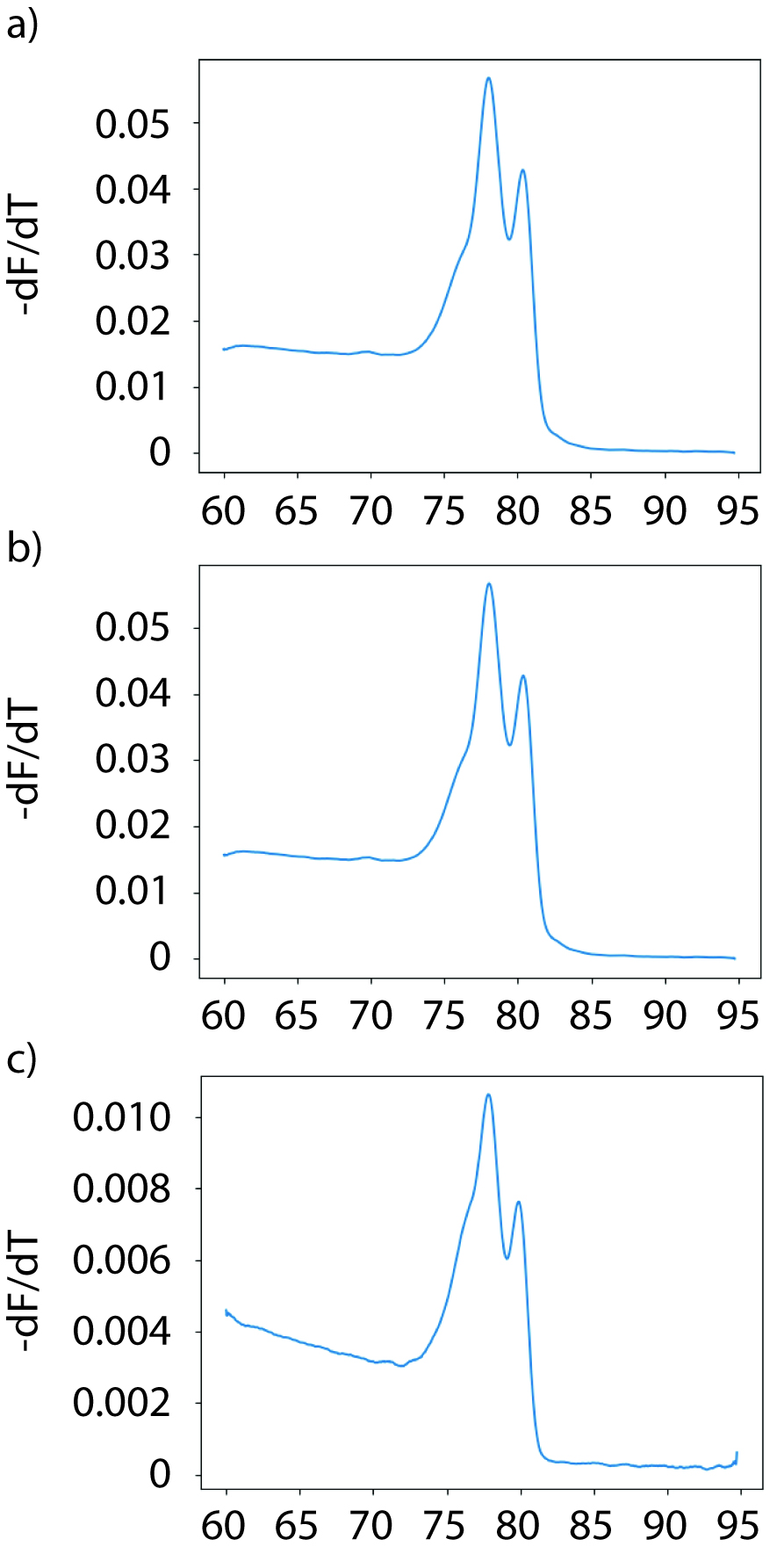

Supplement: Supplementary file 8 — Supplementary Material 8 [file 41598_2025_22370_MOESM8_ESM.jpg]

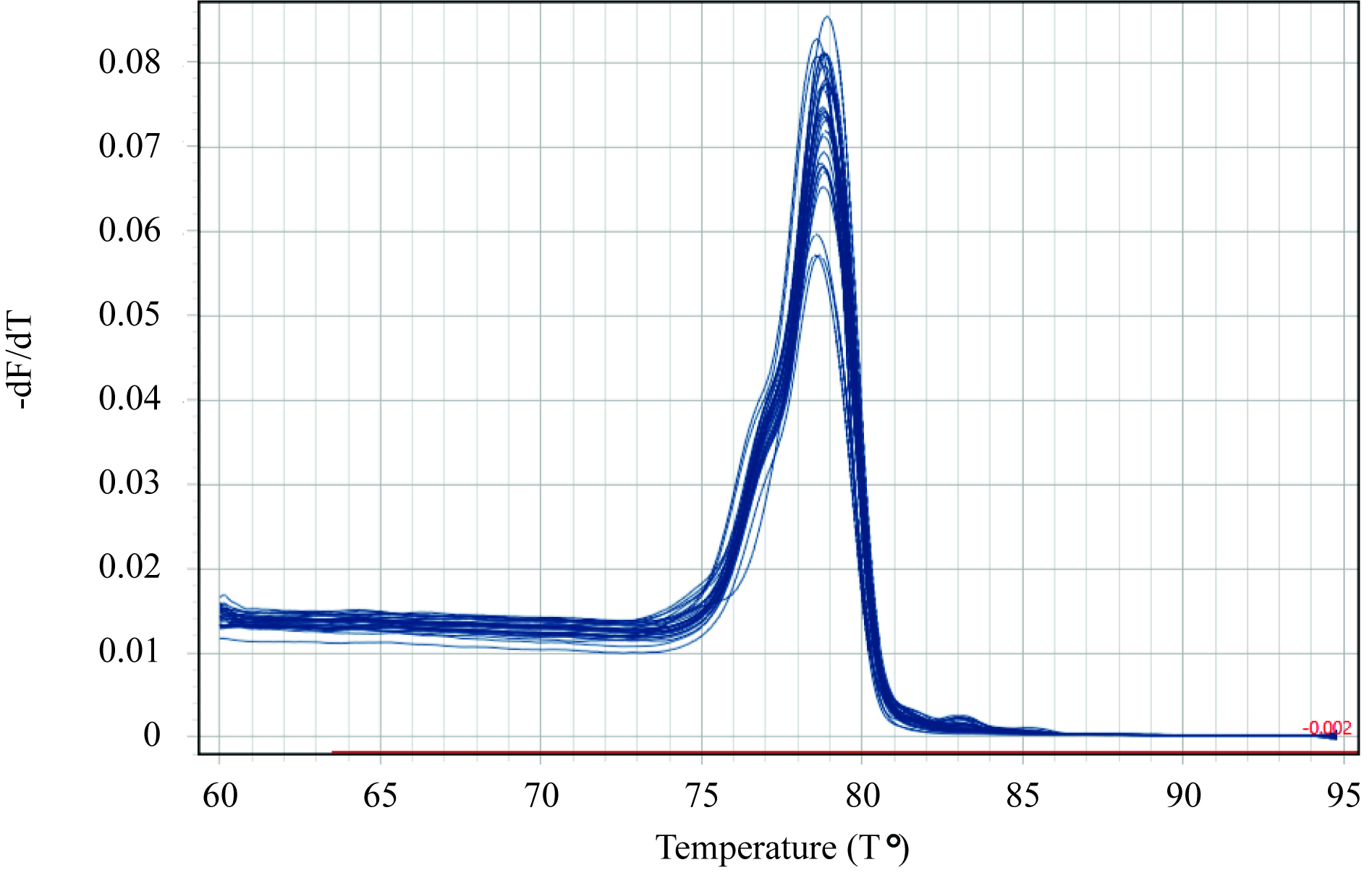

Supplement: Supplementary file 9 — Supplementary Material 9 [file 41598_2025_22370_MOESM9_ESM.jpg]
